# Co-evolution of wetland landscapes, flooding, and human settlement in the Mississippi River Delta Plain

**DOI:** 10.1007/s11625-016-0374-4

**Published:** 2016-05-26

**Authors:** Robert R. Twilley, Samuel J. Bentley, Qin Chen, Douglas A. Edmonds, Scott C. Hagen, Nina S.-N. Lam, Clinton S. Willson, Kehui Xu, DeWitt Braud, R. Hampton Peele, Annabeth McCall

**Affiliations:** 1 Coastal Studies Institute, Louisiana State University, Baton Rouge, USA; 2 College of the Coast and Environment, Louisiana State University, Baton Rouge, USA; 3 Department of Civil and Environmental Engineering, Louisiana State University, Baton Rouge, USA; 4 Center for Computation and Technology, Louisiana State University, Baton Rouge, USA; 5 Louisiana Geological Survey, Louisiana State University, Baton Rouge, LA 70803 USA; 6 Department of Geological Sciences and Center for Geospatial Data Analysis, Indiana University, Bloomington, IN USA; 7 Department of Geology and Geophysics, Louisiana State University, Baton Rouge, LA 70803 USA

**Keywords:** Deltas, Human settlement, Flood risks, Sediment delivery, Wetland loss, Coastal basins

## Abstract

River deltas all over the world are sinking beneath sea-level rise, causing significant threats to natural and social systems. This is due to the combined effects of anthropogenic changes to sediment supply and river flow, subsidence, and sea-level rise, posing an immediate threat to the 500–1,000 million residents, many in megacities that live on deltaic coasts. The Mississippi River Deltaic Plain (MRDP) provides examples for many of the functions and feedbacks, regarding how human river management has impacted source-sink processes in coastal deltaic basins, resulting in human settlements more at risk to coastal storms. The survival of human settlement on the MRDP is arguably coupled to a shifting mass balance between a deltaic landscape occupied by either land built by the Mississippi River or water occupied by the Gulf of Mexico. We developed an approach to compare 50 % *L:W* isopleths (*L:W* is ratio of land to water) across the Atchafalaya and Terrebonne Basins to test landscape behavior over the last six decades to measure delta instability in coastal deltaic basins as a function of reduced sediment supply from river flooding. The Atchafalaya Basin, with continued sediment delivery, compared to Terrebonne Basin, with reduced river inputs, allow us to test assumptions of how coastal deltaic basins respond to river management over the last 75 years by analyzing landward migration rate of 50 % *L:W* isopleths between 1932 and 2010. The average landward migration for Terrebonne Basin was nearly 17,000 m (17 km) compared to only 22 m in Atchafalaya Basin over the last 78 years (*p* < 0.001), resulting in migration rates of 218 m/year (0.22 km/year) and <0.5 m/year, respectively. In addition, freshwater vegetation expanded in Atchafalaya Basin since 1949 compared to migration of intermediate and brackish marshes landward in the Terrebonne Basin. Changes in salt marsh vegetation patterns were very distinct in these two basins with gain of 25 % in the Terrebonne Basin compared to 90 % decrease in the Atchafalaya Basin since 1949. These shifts in vegetation types as *L:W* ratio decreases with reduced sediment input and increase in salinity also coincide with an increase in wind fetch in Terrebonne Bay. In the upper Terrebonne Bay, where the largest landward migration of the 50 % *L:W* ratio isopleth occurred, we estimate that the wave power has increased by 50–100 % from 1932 to 2010, as the bathymetric and topographic conditions changed, and increase in maximum storm-surge height also increased owing to the landward migration of the *L:W* ratio isopleth. We argue that this balance of land relative to water in this delta provides a much clearer understanding of increased flood risk from tropical cyclones rather than just estimates of areal land loss. We describe how coastal deltaic basins of the MRDP can be used as experimental landscapes to provide insights into how varying degrees of sediment delivery to coastal deltaic floodplains change flooding risks of a sinking delta using landward migrations of 50 % *L:W* isopleths. The nonlinear response of migrating *L:W* isopleths as wind fetch increases is a critical feedback effect that should influence human river-management decisions in deltaic coast. Changes in land area alone do not capture how corresponding landscape degradation and increased water area can lead to exponential increase in flood risk to human populations in low-lying coastal regions. Reduced land formation in coastal deltaic basins (measured by changes in the land:water ratio) can contribute significantly to increasing flood risks by removing the negative feedback of wetlands on wave and storm-surge that occur during extreme weather events. Increased flood risks will promote population migration as human risks associated with living in a deltaic landscape increase, as land is submerged and coastal inundation threats rise. These system linkages in dynamic deltaic coasts define a balance of river management and human settlement dependent on a certain level of land area within coastal deltaic basins (*L*).

## Introduction

River deltas all over the world are sinking beneath increasing sea levels, causing significant threats to natural and social systems (Syvitski et al. 2009). This is due to the combined effects of anthropogenic changes to sediment supply and river flow, subsidence, and sea-level rise, posing an immediate threat to the 500–1000 million residents, many in megacities that live on deltaic coasts (Vörösmarty et al. 2009). Compounding the problem, most deltaic coasts are also important regions for agricultural production, fisheries, hydrocarbon production, and global shipments of commercial goods. Solving this problem requires understanding how human settlement patterns and economies have co-evolved with the physical system linked to major river basins (Syvitski and Saito 2007; Vörösmarty et al. 2009; Day et al. 2012; Chen et al. 2012). In the last 100 years, river-management decisions have affected human settlements on most major river deltas, including Nile, Po, Yellow, and Pearl (Syvitski et al. 2009). For instance, river-management projects—designed to stimulate navigation, reduce river flooding, enhance agriculture production and energy exploration, and protect increased human settlement—have had the correlated impact of increasing land loss and the threat of tropical cyclone inundation (Syvitski et al. 2009). Given observed long-term rates of land loss, it is not clear if human occupation on many coastal river deltas is sustainable (Tessler et al. 2015). This article addresses sustainability of deltas and associated human communities by advancing systems analysis that focuses on how historical river engineering decisions have reduced sediment supply, reduced wetland area, increased vulnerability to coastal flooding, and impact human settlement.

**Fig. 1:**
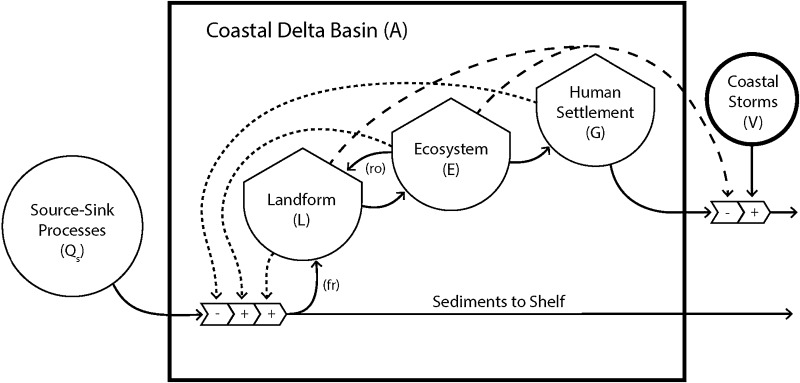
Conceptual diagram of the interactions of source-sink processes of major river basins and delta development, including co-evolution of land formation (*L*), ecosystems (*E*), human settlement (*G*), and flooding risks (*V*). (*r*_*o*_) is the contribution of organic production to land elevation, and (*fr*) is fraction of sediment delivery to coastal zone retained in formation of land

The Mississippi River delta plain (MRDP, Fig. 1) provides examples for many of the functions and feedbacks, regarding how human river management has impacted source-sink processes in coastal deltaic basins, resulting in human settlements more at flood risk to coastal storms (Day et al. 2007; Blum and Roberts 2009). The Mississippi River is one of a select few continental-scale systems that connect the watershed to the coast, representing a conduit for water, sediment, and nutrients delivered to the Gulf of Mexico from a drainage basin of 3.344 × 10^6^ km^2^ (Coleman et al. 1998; Bentley et al. 2015). This expansive watershed is a network of rivers and streams that connect some 41 % of the conterminous United States (USA). The Mississippi River Basin is the fourth largest system in the world in terms of drainage area, and the seventh largest in terms of discharge and sediment load (Coleman et al. 1998; Milliman and Farnsworth 2011). The USA as a whole has a stake in the scientific and societal welfare of the MRDP, with its rich natural and cultural resources, including the infrastructure for the largest port (by tonnage) in the world, 17 % of USA oil and 25 % of USA natural gas production, and a ~$3B per year coastal fishing industry, some 30 % of the USA total (SEST 2012). Wetland, estuarine, and nearshore marine habitats are critical to northern Gulf of Mexico coastal ecosystem function, continental-scale carbon and nitrogen cycling and sequestration, and the economy and lifestyles of the people of Louisiana and USA. This is one of the most highly engineered watershed and delta landscapes in the world, capturing the best and the worst in balancing economic development, national priorities in navigation and agriculture, public safety, and delta sustainability (Galloway et al. 2009; Bentley et al. 2015). The consequences have been the development of several environmental catastrophes at the coastal end of the river basin, where the highest wetland loss rate (Day et al. 2007) and largest hypoxic zone (Rabalais et al. 2002) in North America exists. Now, there is renewed effort in trying to restore the wetlands of the MRDP and to reduce the runoff of excessive nutrients to the Gulf of Mexico (Day et al. 2007; Paola et al. 2011). Such bold actions of restoring natural processes within the river basin are complicated by the availability of a few options that can accommodate the requirement to control river floods, maintain navigation, and promote agricultural production.

Coastal Louisiana, with its wealth of natural resources, has had a long history of humans attempting to manage the flood risks of occupying an extremely dynamic deltaic environment. There are examples of how environmental and social systems have adapted to sea-level rise, subsidence, and hurricanes to accommodate the sustainable development. Wetlands of the MRDP consist of marshes, forested wetlands, and barrier islands that account for 60 % of the coastal wetlands in the lower 48 states of USA (Boesch et al. 1994; Gosselink et al. 1998). Prior to significant human settlement, land building and deltaic processes over the last several thousand years resulted in a net increase of more than 2.5 million ha of coastal wetlands (Coleman et al. 1998). During this time, wetlands were able to expand across the delta even with the occurrence of sea-level rise, subsidence, and hurricanes, as sediment supply was sufficient to accommodate the net effects of a sinking delta. Following human settlement and key decisions on river management that will be described below, this coastal wetland landscape has been degrading for the past 50 years, owing to the fact that wetlands drown when insufficient sediment is supplied to counter relative rise in sea level (Boesch et al. 1994; Day et al. 2007; Paola et al. 2011; Edmonds 2012b). In response, wetland loss in coastal Louisiana increased dramatically over the last 50 years, with losses reaching a peak of around 102 km^2^/year in the mid 1970s (Britsch and Dunbar 1993). Since 1956, almost 4000 km^2^ of Louisiana’s coastal wetlands have been converted to open water (Britsch and Dunbar 1993; Coleman et al. 1998; Barras et al. 2004). The rate of wetland loss has declined over the last two decades, and currently coastal Louisiana is losing about 44 km^2^ of wetlands each year (Barras et al. 2008). It is estimated that an additional 1800 km^2^ of wetlands will be lost from the MRDP by the year 2050 (Barras et al. 2004; Barras 2009). At present, coastal waters have submerged 25 % of this productive delta, as the Gulf of Mexico moves slowly inland closer to human settlements that were developed in their present location nearly 300 years ago.

The survival of human settlement on MRDP is arguably coupled to a shifting balance between a deltaic landscape occupied by either land built by the Mississippi River or water occupied by the Gulf of Mexico. We argue that the balance of land relative to water in this delta will determine how people deal with increased flood risks from tropical cyclones, as this retreating coast experiences rising seas and subsiding landscapes. Ideally, river-management decisions should reduce flooding and promote navigation, while maintaining natural processes necessary to sustain the physical landscape upon which the safety of human settlement is so dependent. We describe how the coastal deltaic basins of the MRDP provide insights into how different river-management strategies, with varying degrees of sediment delivery to coastal deltaic floodplains, change the patterns of how deltaic coasts and human settlement co-evolve. This region of the MRDP has historically been manipulated by river engineering decisions to control river flooding and maintain navigation across a landscape with extensive human settlement, and has recently undergone substantial change in both land area and population. The focus on protecting human settlements from river flooding has lead to feedback effects on sediment distributions that have resulted in increased flood risks from coastal waters. We focus on flood risk reduction by wetland landscapes in the deltaic plain as an ecosystem service linked to river engineering options along deltaic coasts. Understanding the connections among river-management decisions, delta landform evolution, storm-surge risks, and human settlement decisions provide guidance to restoration, protection, and regional planning processes (Twilley et al. 2008). The coastal deltaic basins of the MRDP provide insights into how different river-management strategies, with varying degrees of sediment delivery to coastal deltaic floodplains, change the patterns of how human settlement co-evolve with flooding risks of a sinking delta. This region can be used to demonstrate and explore feedback effects among reducing sediment delivery with human river-management decisions in deltaic coast, corresponding landscape degradation and increased water area that lead to human perception of increased flooding risk (Fig. 1). Such feedback loops are common in deltaic coasts globally (Chen et al. 2012; Tessler et al. 2015), and understanding system behaviors will provide insights on approaches to restoration and protection strategies under a changing climate.

### Evolution of deltas and emergent ecosystems

The formation of deltaic lobes (*L*, Fig. 1) within a coastal basin (*A*, Fig. 1) is built by coastal accumulation of fluvial sediment as part of source-to-sink processes connecting large river basins to continental margins (e.g., Bentley et al. 2015). Newly emerged ecosystems develop on these landforms with specific vegetative patterns (*E*, Fig. 1) as a function of elevation controlled by the self-organization processes of geomorphic features (Paola et al. 2011; Nardin and Edmonds 2014). These coastal deltaic floodplains co-evolve with the geomorphic features forming extensive wetland landscapes along continental margins that prograde out to coastal ocean environments (Gosselink et al. 1998). Patterns of human communities (*G*, Fig. 1) living on deltaic landscapes also follow features of land area (*L*, Fig. 1) within coastal basins, forming a co-evolution of delta and human development, as communities seek higher elevations along natural levees (Davis 2000).

The sustainable land area (*L*) within a coastal deltaic basin (*A*) for a given sediment supply and local relative rise in sea level (accounting for subsidence) is defined by Paola et al. (2011) as:

1 $$L = \frac{{Q_{s} f_{r} \left( {1 + r_{0} } \right)}}{{C_{0} \left( {\sigma + H} \right)}}$$

where *Q*_*s*_ is volumetric sediment discharge, *f*_*r*_ is the fraction of sediment delivered that is retained for land building (Fig. 1), *r*_*0*_ is the fraction of sediment volume contributed by organic production (feedback Fig. 1), *C*_*0*_ is solids volume fraction, *σ* is local subsidence rate, and *H* is the rate of eustatic sea-level rise. The sum of *H* and *σ* represent the relative sea-level rise (RSLR, mm/year) to which landscape surfaces must respond (by vertical accretion) to maintain a constant land area (*L*) in deltaic coastal deltaic basins (*A*). We modified the original equation by Paola et al. (2011) to focus on the temporal and spatial scales within coastal deltaic basins to define significant issues of river management and ecosystem sustainability relative to two-to-three generations of people living in the delta.

A coastal deltaic basin has a composition of land area and water area, depending on the relative supply of sediment (coastal deltaic basin area (*A*) = water area (*W*) + land area (*L*), Fig. 1). As *L* increases with sediment supply sufficient to compensate for RSLR, the land:water (*L*:*W*) ratio of the coastal deltaic basin will increase. If sediment supply in Eq. 1 is insufficient to compensate for RSLR, then *L* will decrease, resulting in corresponding reduction in *L*:*W* ratio. Delta mass balance is defined within Eq. 1 by denoting that there is a rate of sediment supply that compensates for RSLR, given a contribution of organic production to land elevation (*r*_*o*_). This mass balance suggests that there is rate of sediment supply that can sustain a stable *L*:*W* ratio in coastal deltaic basins that represent a measure of delta sustainability. The apparent simplicity of this delta mass balance condition between sediment supply and RSLR, leading to a constant *L:W* ratio, is deceiving, as terms on the right hand side of Eq. 1 are each complicated functions of multiple interacting physical, geological, and ecosystem processes. For example, the effects of sediment supply on land elevation change ecosystem types and these vegetative processes control marsh inputs of organic matter (*r*_*o*_) and sediment retention (*fr*) (Nardin and Edmonds 2014). Thus, both the land (*L*) and ecosystem features (*E*) in Fig. 1 represent positive feedback effects on land area formation in the coastal deltaic basin, contributing to increases in *L:W* ratio. If newly formed land of the MRDP does not receive new sediment from river floods to increase elevation equal to RSLR, then land becomes submerged and reverts back to the sea, resulting in decrease in *L:W* ratios.

**Fig. 2:**
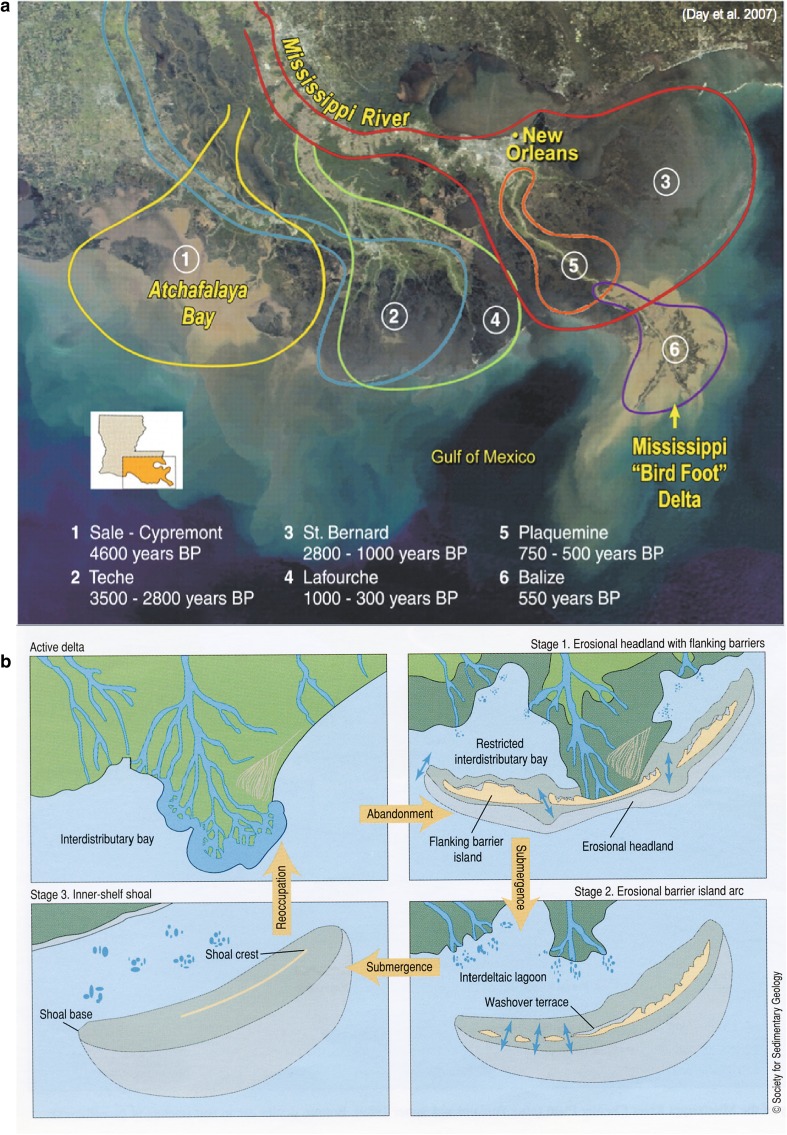
**a** *Upper panel* Map of the Mississippi River Delta Plain (MRDP) based on satellite image overlaid with the historical delta lobe occurrences over the last 7000 years (from Day et al. 2007). **b** *Lower panel* Stages of the delta cycle, including the phases of river occupation, land formation, and ecosystem development, along with river abandonment and wetland loss (from Penland et al. 1988; modified from Gosselink et al. 1998)

The Holocene Mississippi River Delta was mainly formed over the last 7000 years through the processes of delta lobe creation and abandonment (Penland et al. 1988; Boesch et al. 1994; Roberts 1997). Delta switching (Fig. 2a) is the cyclic shifting of the locus of sediment deposition sufficient to build land that can emerge above coastal waters (Roberts 1997). These major delta-building events have occurred every one-to-two thousand years and were characterized by a rapid expansion of new lands extending out from the coast upon which diverse communities of wetlands colonized to form the largest delta in North America (Roberts 1997; Coleman et al. 1998). Maintaining this newly emerged landscape along the deltaic coast of Louisiana requires a continuous supply of new sediment from the Mississippi River as described in Eq. 1 (Paola et al. 2011). Most significant amounts of sediment are delivered to the coast during river floods that deliver mud and sand to wetland floodplains (Roberts 1997; Coleman et al. 1998; Paola et al. 2011). New sediment along the coast from river input can also be distributed in wetlands during frontal storms and cyclones, adding to sediment budget and marsh elevation (Cahoon 2006; Turner et al. 2006; Smith et al. 2015; Roberts et al. 2015). This new sediment adds elevation to the surface of existing deltaic wetlands, which becomes an important process to compensate for the negative effects of sea-level rise and subsidence.

The delta cycle is a fundamental concept that describes this pattern of coastal basin formation and degradation in the provinces of MRDP over 1000–2000 year increments of river avulsions (Fig. 2b; Coleman et al. 1998). The formation of land during fluvial-driven delta progradation (with respect to steady-state conditions of mass balance, or delta maintenance, Eq. 1) contrasts strongly with phases of delta deterioration resulting from decreased sediment flux as function of river abandonment (Fig. 2b). The importance of sediment supply to maintaining landform and wetland ecosystems in coastal deltaic basins is evident when the river abandons a coastal region and moves to another location during river avulsion, characteristic of river abandonment in the delta cycle (Fig. 2b; Penland et al. 1988). As sediment supply decreases in the abandoned coastal deltaic basin, land decreases and water area of the coastal deltaic basin increases, decreasing the *L:W* ratio (Gosselink et al. 1998). Along with subsidence, marine erosional processes rise in importance, reworking sandy sediments to form sandy coastal spits and barrier islands that transgress across the subsiding deltaic plain (Penland et al. 1988). If avulsion results in the reoccupation of a subsiding coastal basin by a river, then the delta cycle restarts landscape progradation, increasing *L:W* ratio, and restoring wetland ecosystems within the coastal deltaic basin (Fig. 2b).

**Fig. 3:**
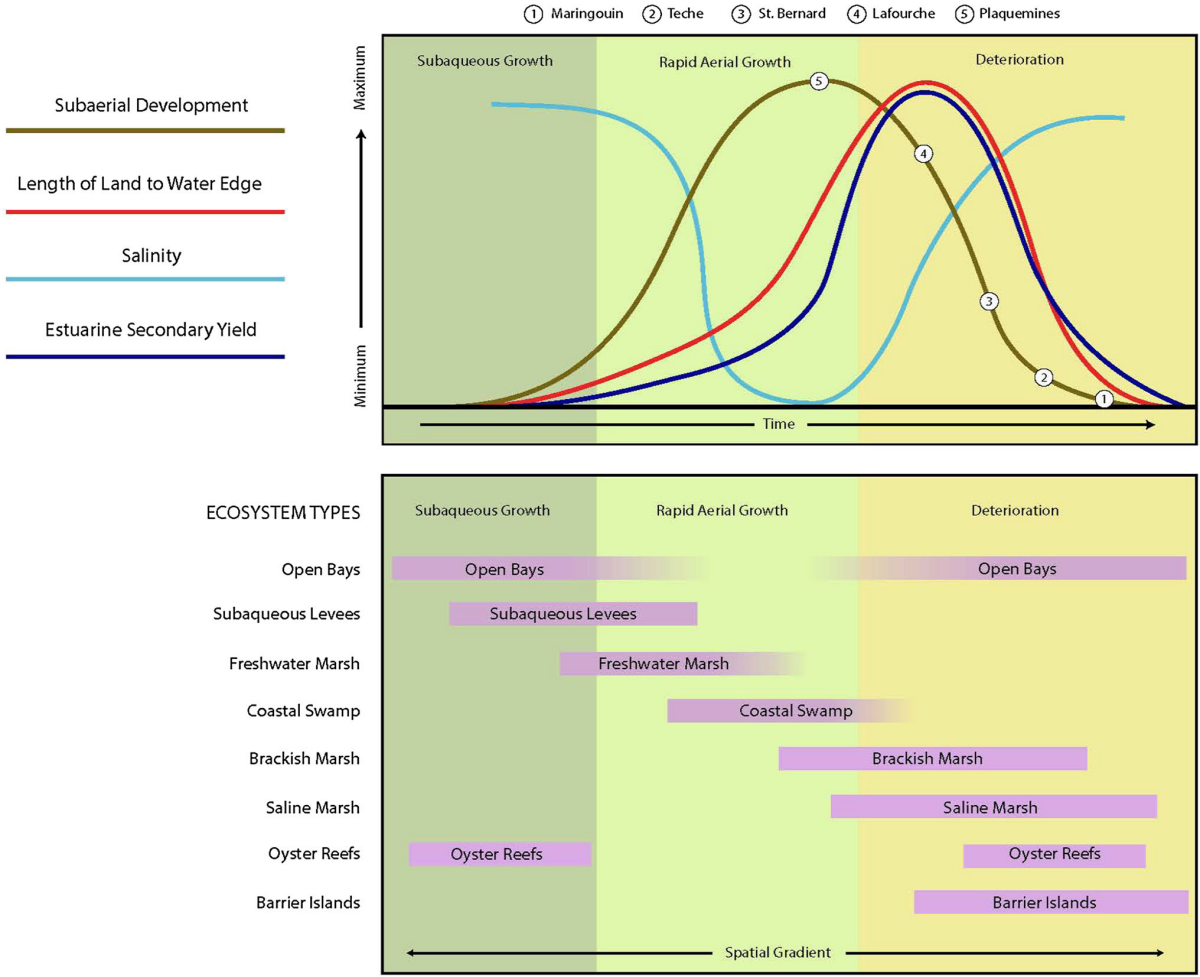
Ecosystem development along the spatial and temporal gradients of delta cycle associated with the magnitude of sediment delivery to coastal deltaic basins, including specific attributes of coastal basins (subaerial development, length of land-to-water edge, salinity, estuarine secondary productivity), and distribution of ecosystem types in a coastal basin with the magnitude of river input (modified from Gagliano and Van Beek 1975; Gosselink et al. 1998). *Numbers* on the subaerial development line correspond to delta lobes in Fig. 2a

This conceptual model of a delta cycle represents the changes in *L* in Eq. 1, as sediment supply (*Q*_*s*_) experiences local pulses driven by river avulsions across specific coastal deltaic basins of MRDP (Fig. 3). If an avulsion results in river abandonment and decrease in sediment input, land decreases as RSLR drowns wetland landscapes in the abandoned basin, *L:W* ratio decreases, and salinity of the coastal basin increases, as salinity isopleths migrate inland (Fig. 3). The distribution of ecosystem types along the delta cycle is driven by the proportion of coastal basin area (*A*) that is land upon which emergent ecosystems colonize modified by the average salinity of wetlands (*E*) and coastal bays (*W*). Thus, the *L:W* ratio and related salinity distributions controlled by the amount of river discharge relative to coastal processes will determine the sources of ecosystem productivity of these coastal deltaic basins (Fig. 3). The balance of processes represented in Eq. 1 at the marsh level determines patterns of land (*L*) and ecosystems (*E*) within a coastal deltaic basin (*A*). In the original derivation of Eq. 1 by Paola et al. (2011), *A* is used to denote the total area of topset of a delta, including land and water components of the area generated by sediment supply (Kim et al. 2009). We will use *A* to denote a fixed area of a coastal deltaic basin as result of Holocene processes in the delta development of MRDP (Fig. 2a), and *L* will denote land area formation and degradation within a coastal deltaic basin as function described in Eq. 1. The focus of this study is on the land area along the coastal shoreline where most of the land area includes wetlands of different types, and water is the bay within a coastal deltaic basin.

### Evolution of deltas and human settlement

Human population change (*G*, Fig. 1) constitutes another indicator of deltaic sustainability. Human migration and relocation is a complex phenomenon. We conceptualize population growth in a delta by a general model (LeSage and Pace 2009; LeSage et al. 2011; Lam et al. 2009, 2012a):

2 $$G_{ij(t)} = f\left( {V_{i(t - 1)} , \, V_{j(t - 1)} , \, B_{i(t - 1)} , \, B_{j(t - 1)} , \, d_{ij} } \right)$$

where *G*_*ij*(*t*)_ represents the population growth rate migrating from place *i* to place *j* at time *t*, *V*_*i*(*t*−*1*)_ is the hazard vulnerability at place *i* at time (*t*−*1*), *B*_*i*(*t*−*1*)_ is the group of variables representing amenities at place *i* at time (*t*−*1*), and *d*_*ij*_ is the distance between origin *i* and destination *j*. Amenities represent social, economic, infrastructure, governmental, and ecological capitals of the place, derived from hazard vulnerability and community resilience research (Cutter et al. 2003, Cutter et al. 2010; Cutter and Finch 2008; Reams et al. 2012; Lam et al. 2014, 2015a, b). One of the major features of human population behavior in coastal deltaic basins is flood risk associated with cyclones in certain coastal zones of the world. While there is an extensive literature on human migration and population dynamical modeling (e.g., Brown and Robinson 2006; Yin and Muller 2007; Niedomysl 2008; Fontaine and Rounsevell 2009), studies on modeling human population dynamics in the context of flood risks and uncertain future climate change impacts are scarce (Li 2015). However, there is recent evidence from coastal zones prone to frequent cyclone disturbance that human migrations are linked to increased risks to flooding during extreme coastal events. Land area and ecosystem features change the wave and storm-surge characteristics of a coastal deltaic basin, such that extensive wetland landscapes are thought to reduce flood risks (Dietsche et al. 2007; Sheng et al. 2012; Medeiros et al. 2012, 2015; Zhao and Chen 2014; Hu et al. 2015; Bilskie and Hagen 2013; Bilskie et al. 2014; Passeri et al. 2015). The potential reduction in flood risks by wetlands produces a positive feedback on human settlement stability (*G*, Fig. 1) by reducing migration associated with coastal storms. Following this argument, land loss or reduction in *L:W* ratio in coastal deltaic basins increases flood risks, which in turn could lead to population decrease.

These two modeling frameworks, one for land area (*L*, Fig. 1) and the other for population migrations (*G*, Fig. 1), suggest that risk of flooding from tropical cyclones in any delta presently represents a significant hazard vulnerability and thus decreases delta sustainability with respect to human communities. Because cyclone surge may be attenuated, as it passes over vegetated land (Dietsche et al. 2007; Sheng et al. 2012; Medeiros et al. 2012, 2015; Zhao and Chen 2014; Hu et al. 2015; Bilskie and Hagen 2013; Bilskie et al. 2014; Passeri et al. 2015), we suggest that the risk (*V*) to communities of cyclone inundation (prior to construction of protection levees) is a function of the extent of deltaic land area (*L*) between the community and open ocean, land elevation (*T*), and ecosystem cover (*E*, including vegetation type):

3 $$V = f\left( {L, T, E} \right)$$

Change in land area in a coastal deltaic basin over the last several decades may have direct impacts on migration rates of coastal human populations through this mechanism, which may be modified by the construction of protection levees. Thus, dynamic deltaic landscapes, both in physical structure (land area, elevation, and vegetation cover) and changes in flood risks (hazard vulnerability), explain how reductions in wetland loss and *L:W* ratio may shape patterns of human settlement, as driven by increased risks of coastal inundation. As stated above, the long history of human behavior in the MRDP to reduce risks to river flooding has also reduced sediment supply leading to reduced land formation in coastal deltaic basins, producing a negative feedback to sustaining land area (*L*, Fig. 1). Thus, reductions in sediment supply (*Q*_*s*_) as a result of river management (construction of protective levees) to reduce risks from river floods can contribute significantly to increase flood risks from coastal storms by removing the capacity of wetlands to reduce wave and storm-surge that occur during extreme weather events. Tradeoffs between river and coastal flood risks will determine population migration as human risks for those living in a deltaic landscape increase, as land is submerged and coastal inundation threats rise. These system linkages in dynamic deltaic coasts define a balance of river management and human settlement dependent on a pattern of environmental succession that sustains a certain level of land area within coastal deltaic basins (*L*) as evidence by constant *L:W* ratio over time.

The impacts of river-management decisions (controlling sediment supply) on both landscape change and flood risks have been understood for over a century, as explained by Corthell (1897) in National Geographic Magazine:

The effect of withholding by the levees from the great areas of the delta of the annual contributions of sedimentary matters, and the steady, though slow, subsidence of these areas, is one which should be considered in deciding the important question of how to protect the people from the flood waters of the river. (Corthell 1897, p. 354).

These statements describe how river-management considerations impact the stability of deltaic wetlands as a function of sediment enrichment from the Mississippi River by compensating for the vertical deficit caused by subsidence. The consequences of the alternative designs considered to manage the Mississippi River during that era were well defined in terms of future risks that would impact human occupation of the deltaic floodplain.

No doubt, the great benefit to the present and two or three following generations accruing from a complete system of absolutely protective levees, excluding the flood waters entirely from the great areas of the lower delta country, far outweighs the disadvantages to future generations from the subsidence of the Gulf delta lands below the level of the sea and their gradual abandonment due to this cause. (Corthell 1897, p. 354).

The risks associated with decisions to manage flood control of the Mississippi River are clearly defined as tradeoffs to the economic benefits associated with opening up the river to navigation and protecting the region’s rich agricultural lands from devastating floods. The economic drivers to minimize flood damages to crops and increase the capacity of commerce along the river were substantial relative to the risks of a delta sinking under the sea, at least for three future generations.

Today, modified deltaic processes (reduced *Q*_*s*_) and altered conditions of the landscape demonstrate that some adjustments to present river-management decisions are critical to allow human settlements and critical infrastructure to safely occupy the MRDP. The fourth generation is now struggling to develop restoration plans within the constraints imposed by the needs to provide river flood control and maintain navigation in the Mississippi River (Barry 1997; Galloway et al. 2009). A goal of the proposed restoration plan, as defined in the Coastal Louisiana Master Plan (Peyronnin et al. 2013), is to build land that will reduce risks to coastal inundation and sustain the economic wealth of this delta region. Diverting freshwater and sediment from the Mississippi River into adjacent coastal wetlands and estuaries is one approach to the comprehensive restoration plan being implemented in Louisiana (Boesch et al. 1994; Day et al. 2007; Twilley and Rivera-Monroy 2009; Paola et al. 2011). The pulsing water-flow of the Mississippi River is believed to be critical for providing sediments necessary to stabilize wetland structure and function in the delta. The manipulation of controlled floods into coastal deltaic basins using river diversion structures may be an important tool for supplying coastal wetlands with freshwater, sediments, and nutrients that can enhance productivity, vertical accretion, and marsh stability. The challenge to implementing such aggressive restoration projects is limited by large-scale testing of how effective such river-management practices will provide critical needs to the fourth generation inhabiting MRDP. We propose that the coastal basins and river engineering practices in the last century provide experimental landscapes to calibrate the outcomes of such projects as way to develop better formulations of how deltas and human settlement co-evolve (Eq. 3).

### Coastal deltaic basins as experimental units of delta instability

**Fig. 4:**
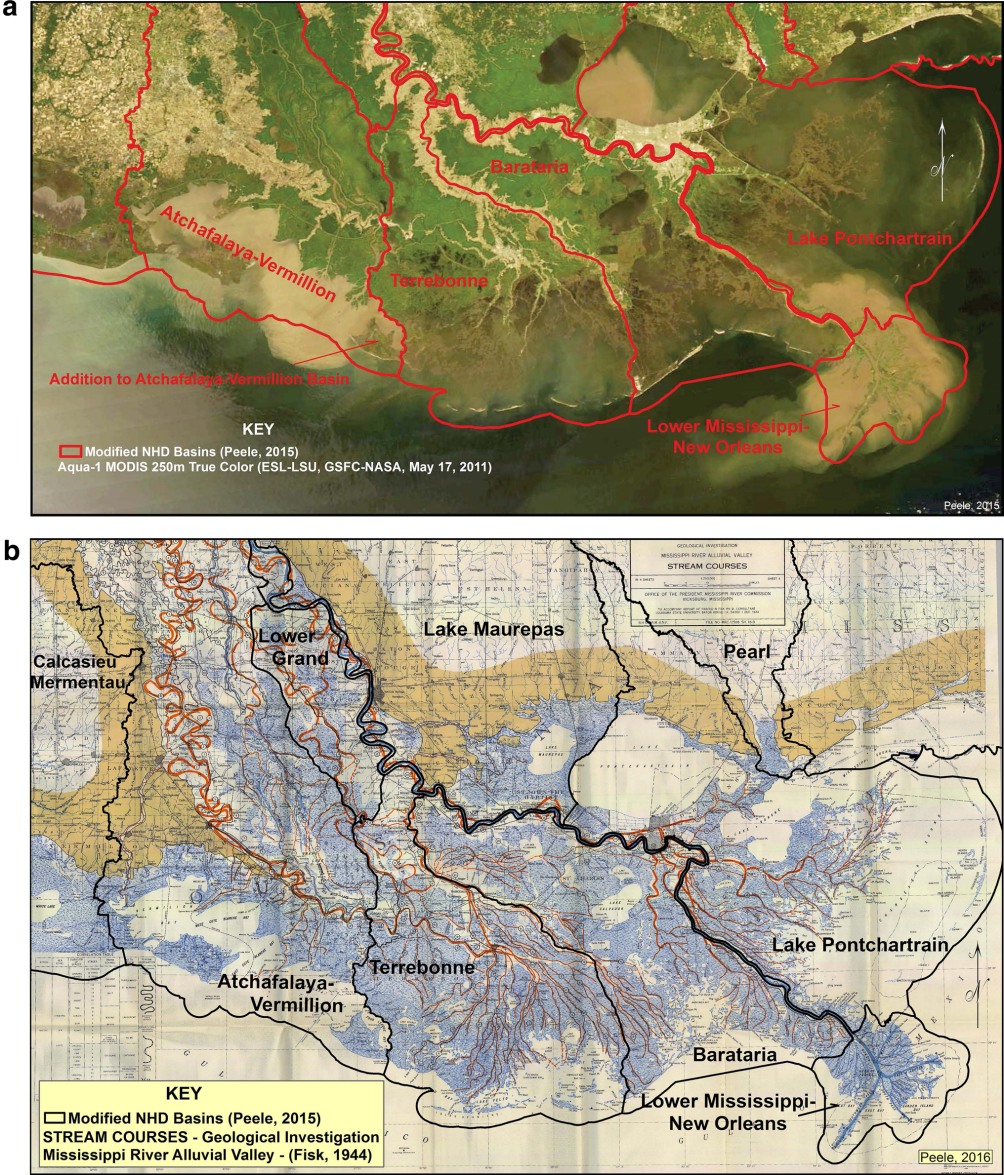
**a** Map of the coastal deltaic basins of Mississippi River Delta Plain (MRDP) based on hydrology overlay on a satellite image of the 2011 flood, showing patterns of sediment plumes at mouth of Mississippi River (east) and Atchafalaya River (west). **b** Map of Mississippi River Delta Plain (MRDP) with historical distributary systems of the Mississippi River as described by Fisk (1944). The original map has been modified to show the location of coastal deltaic basins of MRDP

This process of delta switching of Mississippi River over the last 7000 years has resulted in the formation of six well-defined hydrological coastal deltaic basins today (Fig. 4a). Present coastal deltaic basins in the MRDP represent stages in the delta cycle, as a consequence of sediment supply (*Q*_*s*_), which shape dynamics of land area (*L*) and determine the relative distribution of ecosystems (*E*) colonizing the coastal zone (Figs. 1, 2, 4a; Gagliano and Van Beek 1975; Neill and Deegan 1986; Gosselink et al. 1998). As a distributary channel occupies a coastal deltaic basin, freshwater and sediment will control changes in land formation (subaerial delta area, *L*, Eq. 1) and salinity patterns, which determine landscape patterns in emergent ecosystems (Fig. 3; Shaffer et al. 1992; Holm and Sasser 2001). River-management decisions to promote navigation and provide public safety have been focused on designing river levees and restricting outlets to coastal floodplains, resulting in coastal deltaic basins with varying degrees of sediment delivery. Based on a map developed by Fisk (1944; Fig. 4b), the extensive distributary system of MRDP historically carried sediment to coastal deltaic basins during flood events (Coleman et al. 1998; Roberts and Coleman 1996). As connections between distributaries and Mississippi River became isolated by the formation of locks and levees, coastal deltaic basins have undergone different degrees of river abandonment, as a result of restricted river sediment supply over the last century (Davis 2000). Prior to 1903, two major channels delivered sediment to the central MRDP from Mississippi River: Atchafalaya River (Fig. 4b), and Bayou Lafourche (Fig. 4b, including Bayou Terrebonne from Bayou Lafourche). These two tributaries emptied into Atchafalaya Bay and Terrebonne Bay, respectively (Fig. 4a, b). Prior to closure of Bayou Lafourche, there were estimates that flow during flood-pulsed events was 340 m^3^/s during the 1851 flood (Ellet 1853), compared to flow of about 28 m^3^/s today (CEI 1997). During this same analysis, the amount of river water flowing overbanks from below the mouth of Red River to discharge below New Orleans during the 1851 flood was 3964 m^3^/s of the total flood flow of about 32,125 m^3^/s, or about 12 % of the flood pulse (Ellet 1853). This is the amount of water with sediment that flowed over the riverbanks and through crevasses to coastal deltaic basins nourishing wetlands of the floodplain. River-management decisions over the last century have reduced these fluxes of sediment into wetlands of many of the coastal basins of MRDP.

In contrast to these public work projects that restricted sediment delivery to most of the coastal deltaic basins of the MRDP over the last century, a major connection to river sediment supply was maintained in Atchafalaya Bay, where an outlet with Mississippi River was constructed, known as the Old River Control Structure (Fig. 4a). The Old River Control Structure represents the only location where an outlet has been maintained in the lower deltaic plain of the Mississippi River, emptying water and sediment into Atchafalaya River (along with discharge from Red River) down to Atchafalaya Bay (Roberts 1998; Roberts et al. 2003; Wellner et al. 2005). These flood control structures were designed to provide a floodway to the coast as an “outlet” that would help to protect urban centers downstream at Baton Rouge and New Orleans from flooding conditions. During the 1950s, it became clear that the Mississippi River was slowly migrating to the west and would soon follow the river basin of the Atchafalaya River (Trotter et al.^1998^; Reuss 2004; Edmonds 2012a). The Old River Control Project was designed for 30 % of the combined Mississippi and Red River’s total flow passing down the Atchafalaya River on an annual basis and 70 % down the Mississippi River to New Orleans. Two deltas are currently forming at the mouth of the Atchafalaya River, the Atchafalaya Delta, and the Wax Lake Delta (WLD); however, only the WLD has been allowed to form naturally without any major dredging manipulation. The Atchafalaya Basin is one of the few coastal deltaic basins where land has emerged above mean sea level in the last four decades (the other at mouth of Mississippi River), where subaerial Wax Lake Delta formed after the unusually high spring flood of 1973 (Roberts 1998). Land in this coastal deltaic basin has experienced rapid subaerial growth throughout the last 35 years (1–2 km^2^ year^−1^).

We can consider the Atchafalaya and Terrebonne Basins as experimental coastal deltaic basins to test the concepts described above about how land area (*L*) responds to varying degrees of sediment delivery (*Q*_*s*_) in Eq. 1. The Atchafalaya Basin continuously received sediment, even though structures were built on the Atchafalaya River in 1944–1963 to allow control of water and sediment (Reuss 2004). Terrebonne Basin is an experimental basin where sediment supply from Mississippi River was eliminated in 1903 (LBSE 1904). A satellite image during the 2011 river flood (Fig. 4a) demonstrates present sediment delivery patterns where plumes are evident in Atchafalaya Basin compared to no sediment plume evident in Terrebonne Basin. For reference, the Atchafalaya River discharged 40 Mt/year of sediment in 2008–2010, ~31 % of total Atchafalaya and Mississippi discharge (Allison et al. 2012).

This is the only region of coastal Louisiana that is building deltaic wetlands and confirms the ability of the river to sustain delta landscape if sediment delivery is allowed to occur across the coastal floodplain. Wetlands have colonized the emerging lands of the Atchafalaya and Wax Lake Deltas, including *Sagitarria platyphylla* as the dominant vegetation in the summer and fall. Older lobes of the Wax Lake Delta have a mixed community composed of *Colocasia esculenta*, *Phragmites australis*, *Polygonum punctatum*, *Typha* spp., *Schoenoplectus* spp., and *Zizaniopsis miliacea*. *Salix nigra* is the dominant vegetation present at levees of the older lobes, with an understory of *C. esculenta* and P*. punctatum* (Johnson et al. 1985; Shaffer et al. 1992; Holm and Sasser 2001). Marine and estuarine ecosystems have become less prominent as salinities decrease, and freshwater ecosystems expanded in this coastal deltaic basin, as has been observed in Fourleague Bay over the last several decades (Madden et al. 1988).

**Fig. 5:**
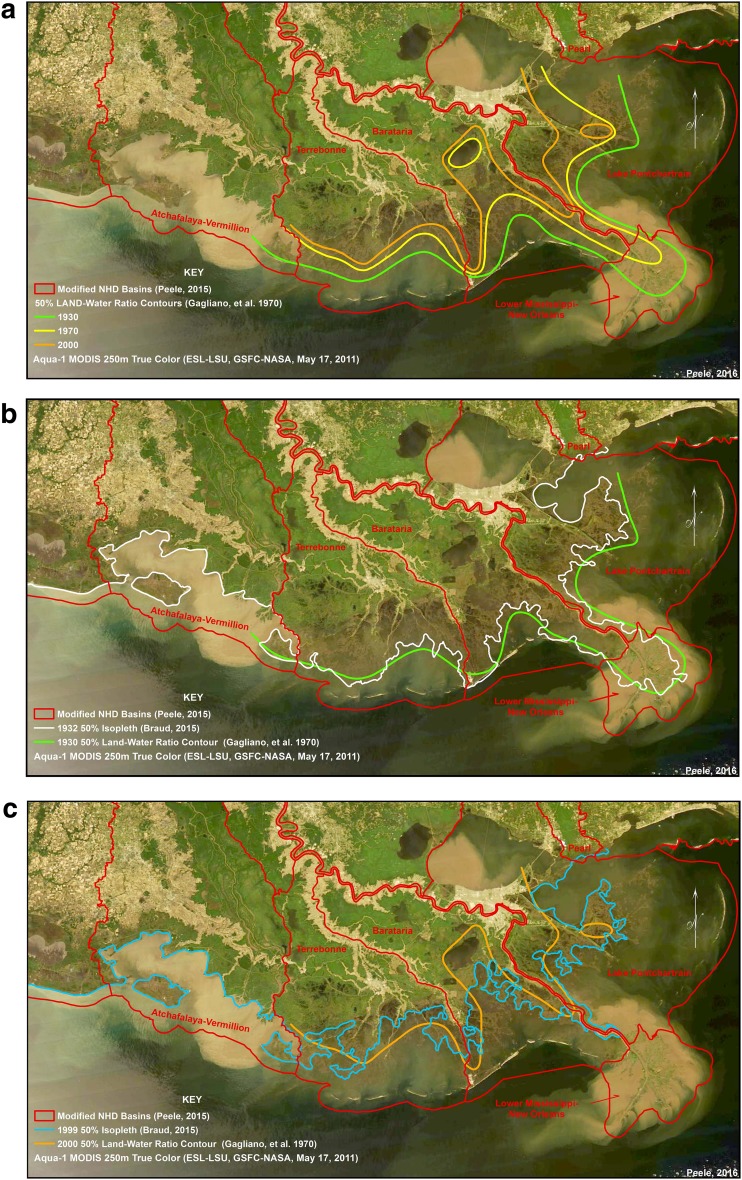
**a** Mississippi River Delta Plain (MRDP) showing progressive position of the 50 % land:water isopleth along the coast (modified from Gagliano et al. 1970). **b** Map of Mississippi River Delta Plain showing isopleths of land:water ratio of 50 % in 1930 (from Gagliano et al. 1970) compared to results of the land:water model of 50 % land:water occurrence of an image in 1932 used in this study. **c** Map of Mississippi River Delta Plain showing isopleths of land:water ratio of 50 % predicted for 2000 (from Gagliano et al. 1970) compared to results of the land:water model of actual image in 1999, showing 50 % land:water isopleth based on model in this study. All isopleths in **a**–**c** have been overlaid upon a satellite image from 2011 showing distribution of sediment during major flood event

The instability of coastal deltaic basins in response to river-management decisions can be tracked by the relative land and water area changes that have occurred over the last 75 years under conditions of sediment delivery, sea-level rise, and subsidence defined in Eq. 1. Gagliano et al. (1970) suggested that plotting isopleths where the *L:W* ratio of 50 % occurred along the coast would indicate the transgression of Gulf of Mexico along MRDP in response to changes in sediment delivery from river management. They plotted an estimate of the 50 % isopleth for 1932 shoreline compared to 1971 map of land and water maps based on the proportion of land and water established for each 7 1/2-minute quadrangle maps between 1930 and 1970, provided by the US Geological Survey. Isopleths were also predicted for 2000 based on wetland loss rates across coastal deltaic basins, showing very strong transgression in coastal basins east and west of Mississippi River. We digitized these estimates by Gagliano et al. (1970) against a satellite image of 2005 (Fig. 5a). These predictions indicate that coastal deltaic basins will experience a reduction of land-to-water, as demonstrated by this landward migration of Gulf of Mexico waters into coastal basin area as a result of wetland loss (Fig. 5a). The actual measurements of decreases in *L:W* ratio (indicated by a movement of the 50 % *L:W* isopleth inland) made in 1932 and 1970 were used to project hypothetical migrations for 2000 by Gagliano et al. (1970). We now have the capability to test these assumptions of how coastal deltaic basins have responded to river management over the last 78 years by analyzing actual migration rates between 1932 and 2010.

We developed a technique to more clearly define the location of the 50 % *L:W* isopleths using query methods of images available in 1932, 1973, 1999, and 2010. A neighborhood moving window operator is applied to a binary land/water raster image of the coastal region derived from aerial or satellite imagery. The function calculates the ratio of land-to-water inside a macro-sized floating analytical processing window and applies the result to the center pixel. The output is a continuous representation of the land–water ratio from which intervals are derived. The *L:W* model shows results across two dated landscapes in 1932 and 1999, with the observations and predictions by Gagliano et al. (1970) overlain upon the results (Fig. 5b, c). The comparison of results from our technique to those of Gagliano et al. (1970) using the 1932 data yields strong similarities (Fig. 5b). This was also the case when comparing results of the 1973 data sets (data not shown). However, the isopleths predicted for 2000 by Gagliano et al. 1970 differ from our analysis of actual migrations based on an image in 1999 (Fig. 5c). Our 50 % *L:W* isopleth has migrated further inland in Terrebonne Basin than predicted by Gagliano, compared to less migration inland from 1970 to 2000 in Barataria Basin. The migration of Gulf of Mexico in Terrebonne Basin shows that the 50 % *L:W* ratio isopleth is nearly 10 km farther inland in 1999 compared to the prediction of 2000 by Gagliano et al. (1970).

**Fig. 6:**
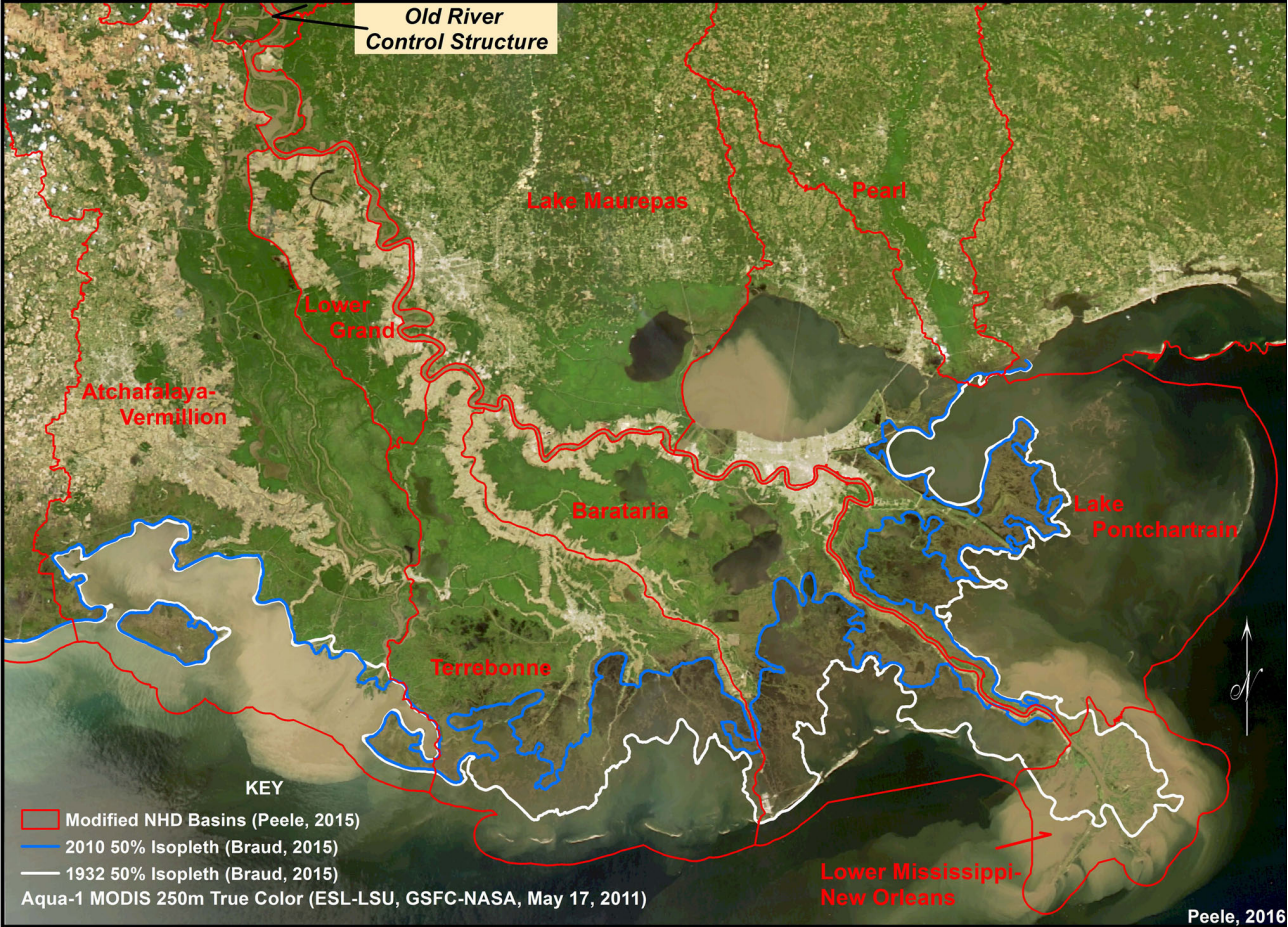
Mississippi River Delta Plain (MRDP) showing progressive position of the 50 % land:water isopleth along the coast across coastal deltaic basins using an image from 1932 compared to image in 2010. Isopleths have been overlaid upon a satellite image from 2011 showing distribution of sediment during major flood event

We extended our analysis of the 50 % *L:W* isopleth across the Atchafalaya and Terrebonne Basins to compare landscape behavior over the last six decades with and without a significant sediment input. By comparing the Atchafalaya Basin, with continued sediment delivery, with Terrebonne Basin, with reduced river inputs, these changes can be used to test models described in Eq. 1. We generated a 50 % *L:W* isopleth for 1932 shoreline image and compared that isopleth to an image in 2010 (Fig. 6). These two isopleths are also compared to a fixed location of the state boundary along the coast for each image. There is very little landward migration of the 50 % *L:W* isopleth in Atchafalaya Basin compared to a clear separation of isopleths between 1932 and 2010 in Terrebonne Basin. We sampled 20 transects perpendicular to the coast in each of the two basins to determine the distance between 1932 and 2010 isopleths (Table 1). Based on these samples, the average landward migration for Terrebonne Basin was nearly 17,000 m (17 km) compared to only 22 m in Atchafalaya Basin over the last 78 years. The difference in these total migration distances using the 20-sampled locations between the two basins was significant at <0.001. We can estimate a landward migration rate of the 50 % *L:W* isopleths in Terrebonne Basin of about 218 m/year (0.22 km/year) compared to <0.5 m/year in Atchafalaya Basin (Table 1; Fig. 6). We also used the total area between the 50 % *L:W* isopleth and the state boundary in both basins in 1932 and 2010 to demonstrate the shifting mass balance between land and water in a basin with and without sediment delivery over the last century. The water areas (area <50 % *L:W*) in the Terrebonne Basin increased 1545 km^2^ compared to only 49 km^2^ in the Atchafalaya Basin during these two images. This is nearly a doubling of area <50 % *L:W* over the last 78 years in Terrebonne Basin compared to <2 % change in Atchafalaya Basin. These area changes represent all landscapes <50 %, so they include less marsh but also the open water component. The key point of this analysis is to establish the rate at which the Gulf of Mexico is actually migrating landward to coastal communities as a function of sediment delivery characteristics.

**Table 1:** Metrics describing the relative change in land-to-water ratios between Atchafalaya and Terrebonne Basins from 1932 to 2010 using the analysis of isopleths that describe the 50 % land:water ratio

Land migration metric	Atchafalaya/Vermilion Basin	Terrebonne Basin
Total migration (m)		
Mean*	22	16,976
SE	224	1852
*N*	20	20
Migration rate (m/year)	0.3	217.6
Area <50 % *L*:*W* (km^2^)		
1932	3019	1804
2010	3068	3349
Difference	49	1545

The migration is based on comparing the 1932 and 2010 isopleths; and the area difference for each year is based on a fixed state boundary for coastal zone

* Means are significantly different at the *p* < 0.001 level

We argue that most of the changes observed between these two experimental basins are due to reduced sediment delivery (*Q*_*s*_ *f*_*r*_) and not differences in subsidence (*σ*) between the two coastal deltaic basins, which could also explain differences in L based on Eq. 1. In general, subsidence rates in our study area decrease inland, as a function of the relative age and thickness of the local deltaic sediments (CPRA 2012). Inland sediments are older and the thickness of a particular deltaic package decreases inland (e.g., Blum and Roberts 2009). These two factors contribute to (but do not entirely control) the observed pattern of inland decrease of subsidence rates. For example, the median subsidence rates for the lower Terrebonne and Atchafalaya basins are 13 ± 6 and 7 ± 3 mm/year, respectively, whereas median subsidence rates for inland Terrebonne and Atchafalaya Basins (mapped as one unit in CPRA 2012) are 6 ± 4 mm/year. Based on this analysis, the inland decrease in subsidence rates should produce an inland deceleration of isopleth migration over time, which is not the pattern we observe. Subsidence certainly contributes to land loss, but in these cases, we propose that sediment supply exerts stronger influence.

**Fig. 7:**
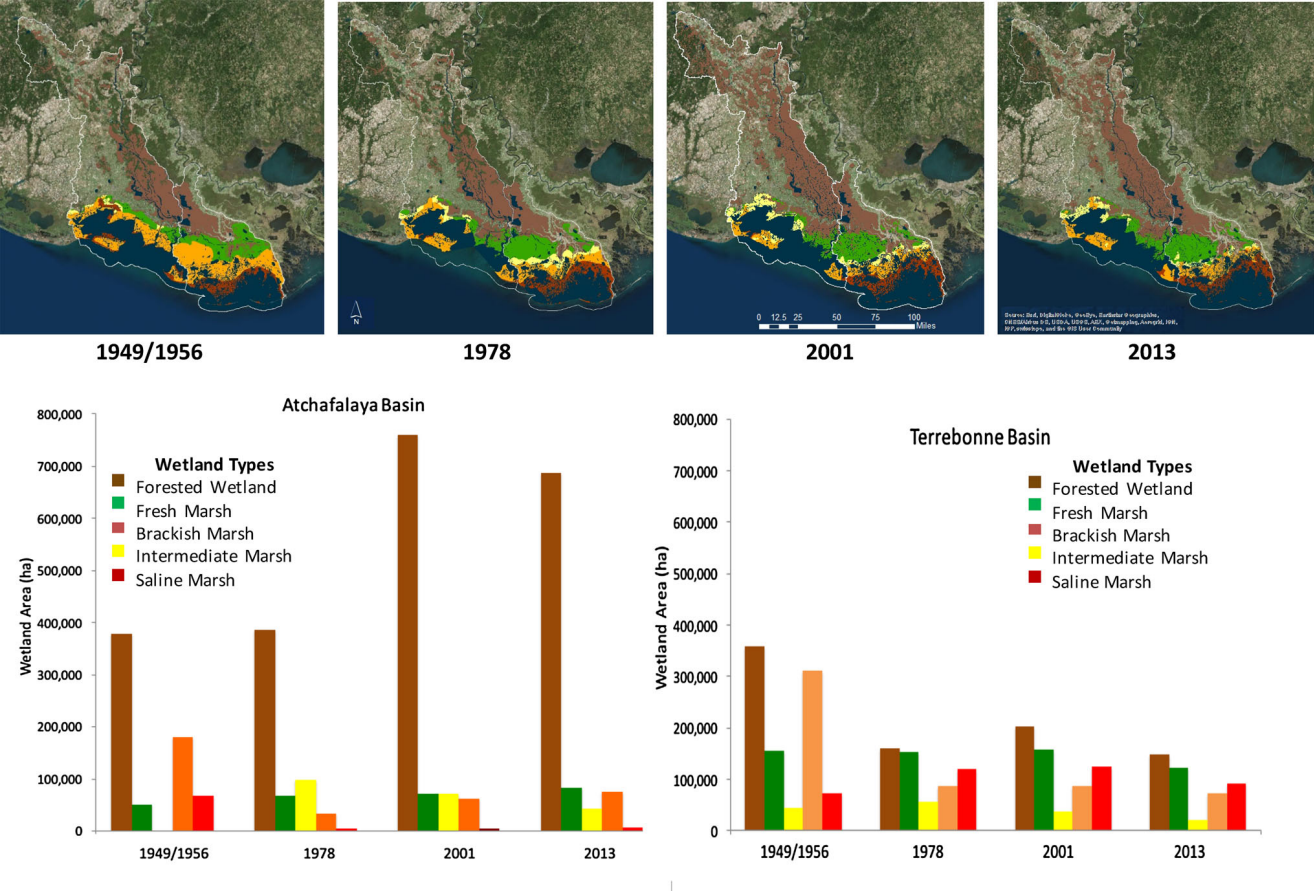
Vegetation maps and wetland distribution for Atchafalaya and Terrebonne Basins from 1949 to 2013 showing shifts in forested wetlands, freshwater marsh, brackish marsh, intermediate marsh, and salt marsh over time in each basin. Description of data sets used: (1) 1949/1956 map (O’Neil 1949, Habitat zones 1956, USGS 1978); (2) 1978 map (Chabreck and Linscombe 1978, USGS 1978); (3) 2001 map (Linscombe and Chabreck 2001, USGS 2003); and (4) 2013 map (Sasser et al. 2014, USGS 2011)

Vegetation change between these two basins also demonstrates how ecosystems (*E*, Fig. 1) co-evolve with the landform area changes (*L*, Fig. 1) between these two experimental basins as a function of river abandonment (Fig. 7). For a comprehensive look at the wetland vegetation zone types throughout the entire Atchafalaya and Terrebonne Basins, existing wetland vegetation surveys and land use/land cover data sets were compiled and analyzed using geoprocessing tools provided in the ArcMap 10.2 software. Existing shapefiles were merged together, combining water boundaries with classified vegetation zones and land use/land cover data. These overlays were clipped to the basin boundaries as defined in Fig. 4a. These maps represent a rough estimation of the extent of wetland vegetation zone dating back to 1949, 1978, 2001, and 2013. Wetland vegetation surveys were conducted throughout the Louisiana coastal zone (Visser et al. 1999). Since the Atchafalaya and Terrebonne Basins boundaries extend beyond the coastal zone, these data sets were combined with the National Land Use/Land Cover data sets, which provide land cover classification for the entire state. These classifications include forested wetland and freshwater emergent wetland. The coastal zone is comprised of the area classified as freshwater emergent wetland in the Land Use/Land Cover data sets. Beyond the coastal zone, most of the wetland vegetation is classified as forested wetland. This allowed for an overlay of the vegetation surveys over the Land Use/Land Cover data sets to develop a full wetland vegetation zone classification for each basin. The land cover classified as water in the Land Use/Land Cover data sets also provides a more accurate representation of water body boundaries throughout the coastal zone during each time period. Water boundaries were also merged with the vegetation zones to calculate a more accurate area.

**Fig. 8:**
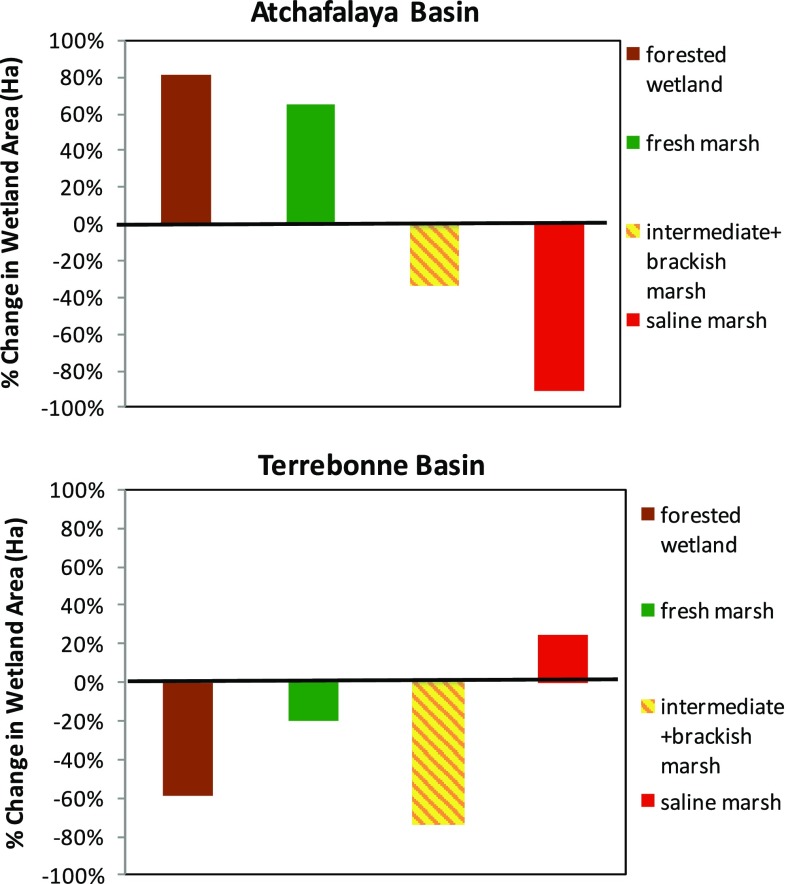
Percent change in the area of four vegetation classes from 1949 to 2013 in Atchafalaya and Terrebonne Basins (the intermediate and brackish marshes have been combined into a single type) using information from maps used in Fig. 7: (1) 1949/1956 map (O’Neil 1949, Habitat zones 1956, USGS 1978); (2) 1978 map (Chabreck and Linscombe 1978, USGS 1978); (3) 2001 map (Linscombe and Chabreck 2001, USGS 2003); and (4) 2013 map (Sasser et al. 2014, USGS 2011)

The Atchafalaya River floodway has a large track of bottomland hardwoods forests that dominated throughout the temporal range from 1956 to 2013, with strong increase from 1978 to 2001. There is less initial area of forested wetlands in the Terrebonne Basin, which actually decreases from 1956 to 1978. It is evident in the maps that a large concentration of freshwater marsh migrates to the mouth of Atchafalaya (including Wax Lake Outlet) from 1956 to 1978, coinciding with the emergence of Wax Lake and Atchafalaya Deltas in the early 1970s. This freshwater band of vegetation expands out from the river in 2001 and 2013. In contrast, the intermediate and brackish marsh systems migrate landward in the Terrebonne Basin. These shifts in vegetation types and cover across these basins are described as a percent of vegetation cover in 2013 compared to 1949. There is an increase of 82 and 65 % for forested wetlands and freshwater wetlands in the Atchafalaya Basin, compared to a loss of about 59 and 21 % for these two vegetation types, respectfully, in Terrebonne Basin. In contrast, the intermediate + brackish marsh area in both basins decreased, 34 % in the Atchafalaya Basin, and 74 % in the Terrebonne Basin (Fig. 8). Salt marsh vegetation patterns were very distinct in these two basins with 90 % decrease in the Atchafalaya Basin since 1949 compared to gain in saltmarsh of 25 % in the Terrebonne Basin (Fig. 8). These shifts in vegetation cover follow predictions based on the delta model (Fig. 3), as *L:W* ratio increases with greater sediment input and decrease in *L:W* as river abandons a coastal deltaic basin.

### Patterns of delta instability and flooding risks

This analysis demonstrates how coastal deltaic basins with and without sediment delivery change land area relative to water area, along with shifts in ecosystem types, in Atchafalaya Basin and Terrebonne Basin, respectively. There was minor landward migration in the *L:W* isopleth from 1930 to 2010, where river floods have not abandoned a coastal deltaic basin. Newly emergent landscapes in the Atchafalaya Basin with *L:W* ratio >50 % appear at the mouth of Atchafalaya River and Wax Lake Outlet, in response to sediment delivery from a river diversion constructed over 50 years ago. As described above, we propose that reduced sediment delivery along Bayou Lafourche that was initiated during the late 1800s and completed in 1903 can explain the landward migration of water from the Gulf of Mexico in Terrebonne Basin. These measurements of *L:W* ratio are indicative of delta instability in coastal deltaic basins as a function of reduced sediment supply from river flooding, as predicted by the delta cycle concept when the river abandons a coastal region.

Environmental succession of coastal deltaic basins linked to river sediment supply describes a gradient in ecosystem services that occur during distinct stages of delta cycle. The succession of these ecosystem services is linked to the ecosystem sequence that occurs as landform, salinity, and elevation change, and relative *L:W* ratio shifts with sediment input. The cumulative measure of ecosystem area and type determines many of the ecosystem attributes in a coastal deltaic basin. For example, at peak *L:W* ratio, salinities are low, and thus mostly freshwater marshes and coastal forests occupy the total area of a coastal basin. Under these conditions, ecosystem services, such as storm-surge reduction is high. In contrast, a coastal deltaic basin with decreasing *L:W* ratio as result of wetland loss and salinity increase is more susceptible to coastal inundation, as forested wetland area declines and water area increases. At some point, the continued reduction in *L:W* ratio increases the risks of flooding with longer fetch lengths that lead to increased wetland erosion and wave formation during extreme weather events that continue to threaten human settlement (Karimpour et al. 2015). These conceptual conditions of ecosystem services during the stages of the delta cycle can be described by the *L:W* ratio that is a function of sediment delivery.

Coastal wetlands, including forests, play an important role in mitigating damages from extreme events, such as tropical storms and hurricanes. Coastal wetlands act as a buffer to protect coastal communities by attenuating strong winds, waves, and storm-surges. The impact of coastal wetlands and forests on storm-surge depends on many factors, such as vegetation properties that lead to resistance to flow of water (e.g., stem/trunk height, rigidity, diameter, density, and coverage) and vegetative properties that lead to reduction of momentum transfer of wind (e.g., canopy, height, density, and coverage), landscape characteristics (e.g., land/water configuration, bathymetry, topography, local geometry, levee, channels, and other features), and storm parameters (e.g., storm track, storm size, duration, forward speed, and wind intensity), as well as the interaction of these factors (Dietsche et al. 2007; Chen et al. 2008; Medeiros et al. 2012; Sheng et al. 2012; Zhao and Chen 2014; Hu et al. 2015; Bilskie and Hagen 2013; Bilskie et al. 2014; Passeri et al. 2014, 2015; Medeiros et al. 2015; Nardin and Edmonds 2014). State-of-the-art numerical models with a realistic representation of land use and land cover (LULC) allow us to quantify the influence of wetlands on inundation of tropical cyclones (Bilskie et al. 2014). Tropical cyclone surge height diminishes with increasing travel distance over land and wetland for a given set of storm and LULC conditions (Bilskie and Hagen 2013). However, small strips of wetlands that effectively attenuate wind waves are insufficient to reduce storm-surge (Chen and Zhao 2012; Jadhav and Chen 2013; Jadhav et al. 2013). Potential benefits of land, wetland, and coastal forests for mitigating flood risks strongly depend on the size of the land area and what is built or growing on it. As shown in Figs. 7 and 8, there is an increase in forested wetlands and freshwater marsh, as sediment supply enriches the Atchafalaya coastal deltaic basin. The former vegetation type has particular influence on storm-surge reduction in this coastal basin; whereas the loss of these habitats in Terrebonne Basin, since 1949, is another factor contributing to increased flooding risk.

**Fig. 9:**
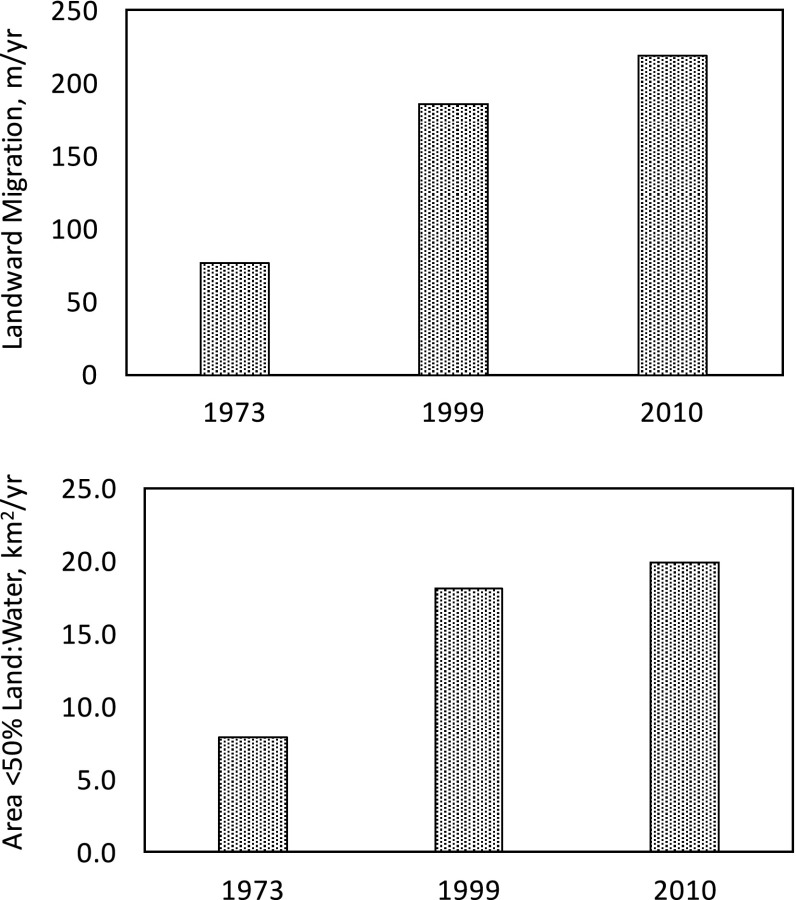
**a** Annual landward migration rates for the 50 % land:water (*L:W*) ratio in Terrebonne Basin based on isopleths in 1932 and difference measured for isopleths calculated from images in 1973, 1999, and 2010. **b** Annual expansion of area <50 % *L:W* (including open water) in Terrebonne Basin based on isopleths in 1932 and difference measured for isopleths calculated from images in 1973, 1999, and 2010

There is some evidence that the *L:W* ratio decrease is nonlinear with time from 1932 to 2010 in Terrebonne Basin (Fig. 9). The landward migration rate from 1932 to 1973 was about 76 m/year, compared to 186 m/year (near 0.2 km/year) by 1999. The landward migration rate based on location of 50 % *L:W* isopleths in 2010 compared to 1932 was 218 m/year. The annual increase in area of *L:W* <50 % in Terrebonne Basin from 1932 to 1973 was 7.8 km^2^/year and increased at proportional levels as migration rates in both 1999 and 2010 (Fig. 9). The conversion of coastal forests to wetlands and wetlands into mud flats not only loses the vegetation-induced drag on winds, waves, and surges, but also allows the surge to amplify in a wider shallow bay (Chen et al. 2008). The increase in annual rates of landward migration as the area of *L:W* <50 % increases demonstrates this acceleration of wetland loss with landward migration of the Gulf of Mexico. Thus, the loss of the same area of wetlands in 2010 should have a greater effect on amplifying flood risks as the fetch of that conversion to water continues to expand the landward migration. Thus, an exponential relationship should exist between flood risks and decreased *L:W* ratio with river abandonment.

As the *L:W* ratio isopleth migrates landward, the wind fetch in Terrebonne Bay increases and the energy dissipation caused by wetland vegetation decreases. Consequently, the wave energy impacting the upland area and the coastal community increases, which can be estimated using the following relationship.

4 $$\frac{{E_{2} }}{{E_{1} }} = \left( {\frac{{F_{2} }}{{F_{1} }}} \right)^{\beta } \left( {\frac{{d_{2} }}{{d_{1} }}} \right)^{\gamma }$$

where *E*_*1*_ and *E*_*2*_ are the wave energy before and after the *L:W* ratio isopleth migrated landward, and *F*_*1*_ and *F*_*2*_ are the corresponding wind fetches, and *d*_*1*_ and *d*_*2*_ are the corresponding average water depths, respectively. Based on the models for wave generation in shallow estuaries (Chen et al. 2005; Karimpour and Chen 2015), it is determined that the exponents in Eq. 4 are as *β* ≈ 0.35–0.5 and γ ≈ 0.8. In the upper Terrebonne Bay, where the largest landward migration of the *L:W* ratio isopleth occurred, we estimate that the wave power has increased by 50–100 % from 1932 to 2010 as the bathymetric and topographic conditions changed.

Similarly, the increase in the maximum storm-surge height owing to the landward migration of the *L:W* ratio isopleth can be estimated by Eq. 5 (Chen et al. 2008).

5 $$\frac{{H_{2} }}{{H_{1} }} = \left( {\frac{{d_{2} }}{{d_{1} }}} \right)\frac{{\sqrt {1 + 2A_{2} } - 1}}{{\sqrt {1 + 2A_{1} } - 1}}$$

where *H*_*1*_ and *H*_*2*_ are the maximum surge height above the water level at the entrance of the estuary before and after the *L:W* ratio isopleth migrated landward, respectively. The factor *A*_*i*_ (*i* = 1, 2) in Eq. 5 reads

6 $$A_{i} = \frac{{n \tau_{s} F_{i} }}{{\rho gd_{i}^{2} }}$$

where *n* = 1.15–1.3, $\tau_{s}$ is the wind stress acting on the water surface, $\rho$ is the water density, and *g* is the gravitational acceleration. Similarly, the increase in the maximum storm-surge height owing to the landward migration of the *L:W* ratio isopleth can be estimated by Eq. 5.

We propose that human population dynamics also respond to these landform changes, such that as *L:W* ratios decrease migrations due to increased flood risk increase (Fig. 1). Our hypothesis is that population decreases with increasing flood risks associated with decreases in *L:W* ratio in coastal deltaic basins. When the risks are greater than the benefits (amenities), people will move away from the region, and the community will become unsustainable. In other words, if there is an increase in flood risks due to the lack of sediment (which leads to a decrease in *L:W* ratio), then populations are expected to decline, and vice versa. We can test this hypothesis by comparing patterns in Atchafalaya Basin and Terrebonne Basin, treating them as experimental coastal deltaic basins with and without sediment delivery. It will also be very useful to understand the conditions when the hypothesis will not hold, such as why some areas have an increase in flood risks but population remains constant or increases, whereas other areas have no increase of flood risks, but population keeps declining.

**Fig. 10:**
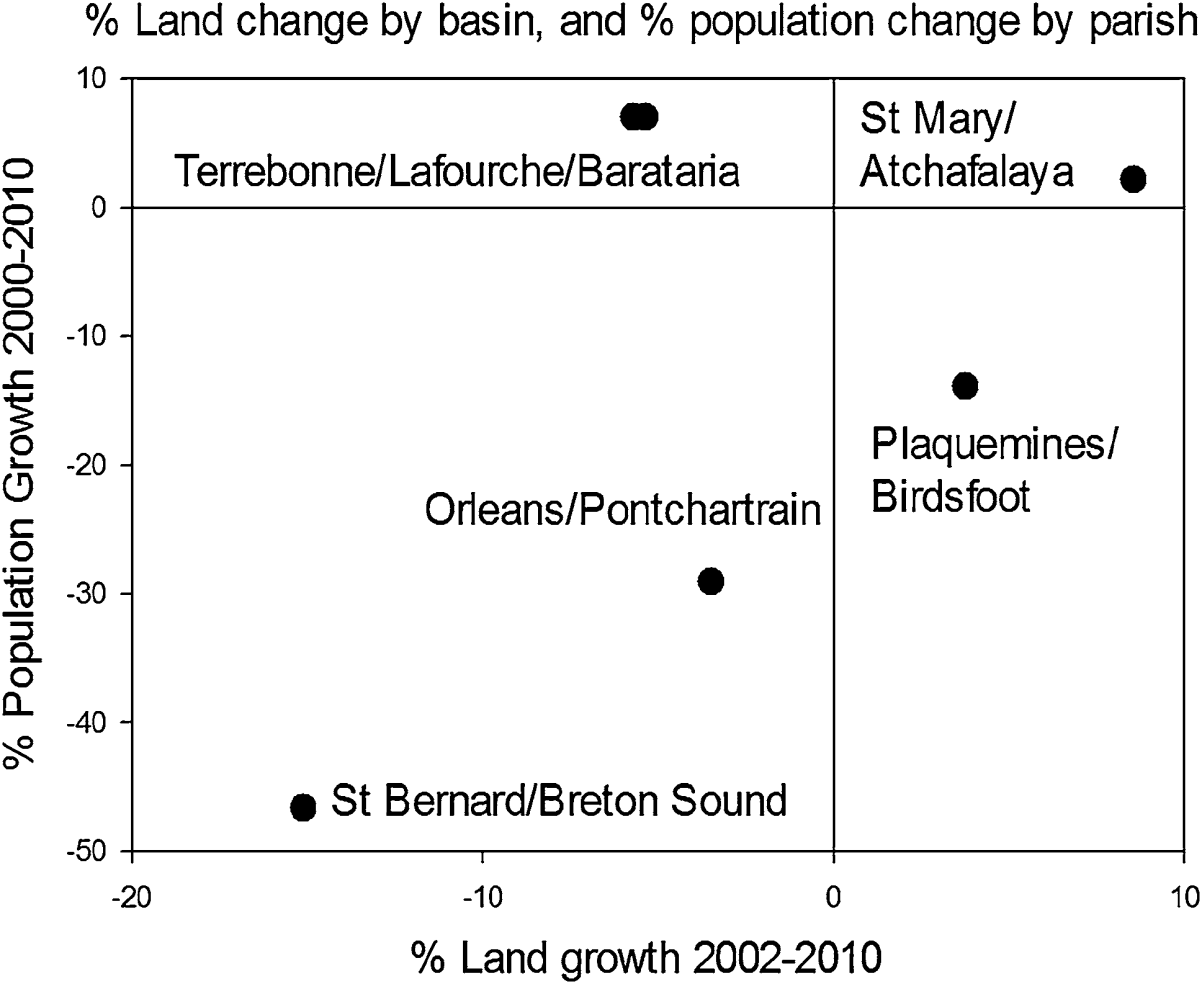
Changes in land area and population by coastal drainage basin (Terrebonne, Barataria, Atchafalaya, Pontchartrain, Birdsfoot, and Breton Sound Basins) and coastal parishes contained within those basins (Terrebonne, Lafourche, St. Mary, Orleans, Plaquemines, and St. Bernard, respectively). Land data are from Couvillion et al. 2011, and population data are from Louisiana State Census Data Center, 2011

There is evidence in the MRDP that humans migrate away from deltaic landscapes experiencing land loss due to increased flooding risks with cyclonic storm-surges (Fig. 10). The two experimental coastal deltaic basins, Atchafalaya Basin and Terrebonne Basin, also offer the possibility of understanding social responses (e.g., population change) to land change and coastal flooding risks. Like communities on many other river deltas worldwide, communities in the central MRDP have deep cultural roots and strong interest in avoiding relocation (Neef et al. 2006; SEST 2012). Population changes in such a highly vulnerable coastal environment may be due to a combination of factors (Li 2015). However, there is evidence that changes in populations in central MRDP are presently occurring (both increases and decreases), and appear to be correlated to rates of land loss and gain (Fig. 10). Two patterns are apparent. First, a nearly linear trend exists for St Mary/Atchafalaya, Plaquemines/Modern Mississippi Birds Foot Delta, Orleans/Pontchartrain, and St Bernard/Breton Sound, suggesting that population change in these regions is linked to decrease of land area. The positive change in land area of St. Mary/Atchafalaya represents our control coastal deltaic basin (Atchafalaya Basin), where sediment supply has existed for several hundred years. Second, Terrebonne and Lafourche parishes in the Terrebonne and adjacent Lafourche coastal basins appear to be maintaining population growth, despite sustained land loss. This contradicts the idea that reduced land area in deltaic coasts would change human settlement with increased risks of flooding. It is possible that Terrebonne and Timbalier regions are influenced by factors not present in the other parish statistics displaying population loss with land loss. The size and productivity of local economies associated with oil and gas industry and extent of levee protection from hurricane flooding may act to sustain populations. The two other regions with reduced land area, Orleans and St. Bernard parishes, have structural protection to reduce risks to coastal storm-surge that failed during Hurricane Katrina, resulting in large extirpation of populations in both parishes (Qiang and Lam 2015, 2016).

Based on our model of land and human co-evolution (Fig. 1) the reductions in delta landscape area (*L*, Eq. 1) in Terrebonne Bay over the last 78 years have caused population decrease due to increased risks from storm-surge as *L:W* ratios decrease. We provide evidence that land loss response to changes in *Q*_*s*_ in coastal shorelines leads to proportional changes to increased storm-surge risks by increased RSLR (investigating both ‘*H*’ and ‘σ’, Eq. 1), as wetlands are drowned, migrate landward, and forested wetlands are lost due to encroachment of humans and the built environment. This is the case in much of Terrebonne Bay, compared to Atchafalaya Bay, where wetlands are maintained by sufficient *Q*_*s*_ and r_o_, potentially diminishing storm-surge risks. We argue that this balance of land relative to water in this delta provide a much clearer understanding of increased flood risk from tropical cyclones rather than just estimates of areal land loss. The coastal deltaic basins of the MRDP can be used as experimental landscapes to provide insights into how varying degrees of sediment delivery to coastal deltaic floodplains change flooding risks of a sinking delta using landward migrations of 50 % *L:W* isopleths. The nonlinear response of migrating *L:W* isopleths as wind fetch increases is a critical feedback effect that should influence human river-management decisions in deltaic coast (Fig. 1). Changes in land area alone do not capture how corresponding landscape degradation and increased water area can lead to exponential increase in flood risk to human populations in low-lying coastal regions. Reduced land formation in coastal deltaic basins (measured by changes in the land:water ratio) can contribute significantly to increase the flood risks by removing the negative feedback of wetlands on wave and storm-surge that occur during extreme weather events. Increased flood risks will promote population migration as human risks associated with living in a deltaic landscape increase, as land is submerged and coastal inundation threats rise. These system linkages in dynamic deltaic coasts define a balance of river management and human settlement dependent on a certain level of land area within coastal deltaic basins (*L*).

## Conclusions

Models of delta-human landscapes need to focus on integrating models of deltaic morphodynamics, wetland ecology, and storm-surge dynamics with human population risks to quantify how decreases in sustainable land area in deltaic coasts influence population dynamics (Chen et al. 2012; Li 2015). Such modeling needs to understand if there are thresholds in land loss in deltaic landscapes (*L*, Eq. 1) that may shift the significance in how flooding risks are realized by human populations (V, Eq. 2). We expect thresholds will exist, whereby, as the *L:W* ratio decreases, there is corresponding increase in fetch resulting in larger tidal surges and wave heights causing nonlinear increase in wetland edge erosion (Mariotti and Fagherazzi 2013; Karimpour et al. 2015). This physical feedback has an impact on the human system, because land loss also produces responses in ‘*G*’ (Eq. 2) along deltaic coasts. There is greater awareness of flooding risks, as coastal deltaic basins lose land coincident with an increase in water area, resulting in increased costs for protection and flood risk mitigation (taxes, insurance, and levee construction). In deltaic coasts around the world, these interactions are compounded by how subsidence (*σ*) and climate change (through H) will threaten sustainable landscapes (Syvitski and Saito 2007), infrastructure, and coastal communities in the future.

Collectively, these issues facing deltaic coastlines have been termed the multi-trillion dollar problem that will have global impacts to public safety, trade, and regional wealth (Foufoula-Georgiou et al. 2011). The combination of reduced sediment supply and increased subsidence, along with the predictions for accelerated sea-level rise, forecast a challenging situation for continued human settlement in most deltas around the world (Tessler et al. 2015). Major investments in engineering strategies and human adaptations, including migrations, are urgently needed given the nonlinear changes in these processes as *L:W* ratio decreases in these deltas, as indicated in this study for the MRDP. The economic consequences of no action or status quo are unimaginable, but real given the patterns that have been observed today for major deltas that can be used to define future scenarios of risks (Tessler et al. 2015). MRDP is used as an example of how engineering capacity may be able to reduce risks in future scenarios, but given factors that may reduce investment effectiveness (such as costs associated with energy constraints), ‘management strategies that address the drivers of RSLR, particularly sediment supply and deposition, will be a core determinant of long-term sustainability over the next century’ (Tessler et al. 2015). The comparative response of Atchafalaya and Terrebonne Basins in this study reflects the boundaries of such future responses of deltas, given the engineering of Mississippi River at Old River Control Structure has stabilized this region of the MRDP. In comparison, the increased coastal flooding investments are required in the Terrebonne Basin to compensate for the lack of sediment supply to reduce risks of RSLR associated with landward migration of Gulf of Mexico. The behavior of these coastal deltaic basins to engineering, river sediment supply, and coastal risks will continue to demonstrate the comparative effectiveness of river management and coastal defenses that will control human settlement patterns and investments in the future. These contrasts in strategies will provide insights to similar situations along deltaic coasts around the world.
